# Folate Deficiency Could Restrain Decidual Angiogenesis in Pregnant Mice

**DOI:** 10.3390/nu7085284

**Published:** 2015-08-04

**Authors:** Yanli Li, Rufei Gao, Xueqing Liu, Xuemei Chen, Xinggui Liao, Yanqing Geng, Yubin Ding, Yingxiong Wang, Junlin He

**Affiliations:** Laboratory of Reproductive Biology, School of Public Health, Chongqing Medical University, Chongqing 400016, China; E-Mails: colourful0324@yeah.net (Y.L.); gao_ru_fei@163.com (R.G.); liuxueqing_cqmu@126.com (X.L.); shirly-cxm@163.com (X.C.); xingjin19880304@163.com (X.L.); aqing000@163.com (Y.G.); dingyb@gmail.com (Y.D.); wyx61221@aliyun.com (Y.W.)

**Keywords:** folate deficiency, decidual angiogenesis, reproductive hormone

## Abstract

The mechanism of birth defects induced by folate deficiency was focused on mainly in fetal development. Little is known about the effect of folate deficiency on the maternal uterus, especially on decidual angiogenesis after implantation which establishes vessel networks to support embryo development. The aim of this study was to investigate the effects of folate deficiency on decidual angiogenesis. Serum folate levels were measured by electrochemiluminescence. The status of decidual angiogenesis was examined by cluster designation 34 (CD34) immunohistochemistry and the expression of angiogenic factors, including vascular endothelial growth factor A (VEGFA), placental growth factor (PLGF), and VEGF receptor 2 (VEGFR2) were also tested. Serum levels of homocysteine (Hcy), follicle stimulating hormone (FSH), luteinizing hormone (LH), prolactin (PRL), progesterone (P4), and estradiol (E2) were detected by Enzyme-linked immunosorbent assay. The folate-deficient mice had a lower folate level and a higher Hcy level. Folate deficiency restrained decidual angiogenesis with significant abnormalities in vascular density and the enlargement and elongation of the vascular sinus. It also showed a reduction in the expressions of VEGFA, VEGFR2, and PLGF. In addition, the serum levels of P4, E2, LH, and PRL were reduced in folate-deficient mice, and the expression of progesterone receptor (PR) and estrogen receptor α (ERα) were abnormal. These results indicated that folate deficiency could impaire decidual angiogenesis and it may be related to the vasculotoxic properties of Hcy and the imbalance of the reproductive hormone.

## 1. Introduction

Birth defects or congenital anomalies are one of the major causes of disability in developed and developing countries [1]. The March of Dimes estimated that 7.4 million infants are born each year with a serious birth defect. Of these births, 94% occur in middle- and low-income countries [2]. Birth defects are also a population health problem affecting the quality of the birth population in China. In high-prevalence areas of China, the prevalence of birth defects was 537.2 per 10,000 births and the first five main birth defects were anencephaly, congenital heart diseases, spina bifida, hydrocephaly, and encephalocele [3]. The causes of birth defects can be grouped into three main categories: (1) genetic, (2) environmental, and (3) complex genetic and unknown. Environmental causes are estimated to be responsible for approximately 5%–10% of the total amount of birth defects. Environmental causes include nutritional deficiencies, maternal illnesses, infectious agents, and teratogenic drugs [2].

Folate is one of the B vitamins and it plays a crucial role in one-carbon metabolism for physiological DNA synthesis and cell division, as well as in the conversion of homocysteine (Hcy) to methionine and so on. The influence of folate nutritional status on pregnancy outcomes has long been recognized [4]. Folate supplementation has been shown to reduce the occurrence of neural tube defects [5]. A preventive effect of folate on heart defects in newborns has also been proposed [6]. Folate deficiency or attendant elevated levels of homocysteine have been associated with orofacial clefts [7], down syndrome [8], placental abruptions [9], pre-eclampsia [10], spontaneous abortion [11], intrauterine growth retardation, and pre-term birth [12]. However, less is known about the mechanism of how folate deficiency induces birth defects. Our previous study showed that folate deficiency did not influence embryo implantation in mice [13]. It suggested that the pregnancy abnormalities, including lower female fertility, lower embryo number, and lower fetal viability, caused by folate deficiency may occur mainly after embryo implantation. Endometrial decidualization, placentation, and the development of the embryo itself are all important for normal pregnancy outcomes after embryo implantation. There have been many studies focused on the effect of folate deficiency on placentation and the development of the embryo itself, whereas little is known about the role of folate deficiency on maternal decidualization [7,8,9,10,11,12]. Angiogenesis, which establishes a network of vessels and sinusoids to support embryo development, is essential for endometrial decidualization [14]. Decidual angiogenesis forms a new vascular network that serves as the first exchange apparatus between the maternal circulation and the developing embryo and thus is a crucial and fundamental process for embryonic survival and a successful pregnancy [15]. Vascular endothelial growth factor A (VEGFA), placental growth factor (PLGF), and vascular endothelial growth factor (VEGF) receptor 2 (VEGFR2) are key molecules regulating decidual angiogenesis and maternal spiral artery remodeling. They are expressed in the endometrium, decidua, and placenta and play an important role in ensuring a successful pregnancy [16,17]. However, it is not known whether the establishment of the decidual vascular network is influenced by folate deficiency. 

Here, we investigated the effect of folate deficiency on decidual angiogenesis after implantation with a folate-deficient pregnant mouse model. This study will help to elucidate the mechanisms of pregnancy abnormalities induced by folate deficiency.

## 2. Experimental Section 

### 2.1. Animals

A folate-deficient pregnant mouse model was established as described by Gao *et al.* [13]. Six-to-eight-week-old National Institutes of Health female mice (mean ± standard error of the mean (SEM): 22 ± 1.8 g) were purchased from the Animal Facility of Chongqing Medical University, China (Certificate No.: SCXK (YU) 20070001) and caged in a specific pathogen-free animal room under a controlled environment (12 h light/12 h darkness). Mice in the folate-deficient group were fed a diet containing no folate (Research Diets, New Brunswick, USA) for five weeks before mating, and mice in the normal group were fed a normal diet. The estrus mice were mated with fertile males of the same strain (E1 = the day a vaginal plug was found). All animal procedures were approved by the Ethics Committee of Chongqing Medical University (NO. 20110016). Pregnant dams were sacrificed on Embryonic day 6 (E6) , Embryonic day 7 (E7), and Embryonic day 8 (E8) at 9–10 a.m and decidual tissue was collected for the subsequent experiments. There were at least 15 mice sacrificed every day in every group.

### 2.2. Detection of Serum Folate, Hcy, P4, E2, FSH, LH, and PRL

Blood samples were collected from the eye socket and placed at room temperature for 3 h to obtain the serum. To verify the utility of the mouse model, serum folate levels of mice were measured using an electrochemiluminescence before mating [18]. The serum levels of Hcy, progesterone (P4), estradiol (E2), follicle stimulating hormone (FSH), luteinizing hormone (LH), and prolactin (PRL) were detected using an enzyme-linked immunosorbent assay (ELISA) (Yan Hui Biological Technology, Shanghai, China) according to the manufacturer’s recommended instructions.

### 2.3. Hematoxylin-Eosin (H&E) Staining

The uterus was extracted and fixed in 4% paraformaldehyde (PFA) solution overnight at 4 °C and then embedded in paraffin. Tissue sections (4 μm) were mounted on glass slides. H&E staining was performed using standard protocols on the paraffin sections. The sections were mounted and stained with hematoxylin-eosin. A minimum of 10 histological sections from the uteri were assessed using 10× or 40× magnification and photographed using an Olympus B × 50 (Olympus) photomicroscope.

### 2.4. Immunohistochemistry

The uterus was extracted and fixed in 4% PFA solution overnight at 4 °C and then embedded in paraffin. Tissue sections were deparaffinized in xylene and rehydrated in descending concentrations of ethanol, followed by antigen retrieval in an Ethylene Diamine Tetraacetic Acid Antigen Retrieval Solution (pH 8.0, Beyotim, Shanghai, China) for 20 min in a microwave oven at 95 °C. Endogenous peroxidase was inhibited by incubation with 3% H_2_O_2_ for 10 min at room temperature. The tissue sections were blocked in 10% normal goat serum for 30 min. The sections were incubated with a rabbit monoclonal anti-cluster designation 34 (CD34) (ab81289, Abcam, Shanghai, China) antibody at a 1:100 dilution, a rabbit monoclonal anti-VEGFA (ab52917, Shanghai, China) antibody at a 1:50 dilution, a rabbit polyclonal anti-VEGFR2 (07-158, Millipore, Billerica, USA) antibody at a 1:100 dilution, and a goat polyclonal anti-PLGF (sc-1882, Santa Cruz, California, USA) antibody at a 1:30 dilution at 4 °C overnight. After incubation with the primary antibody, the tissue sections were incubated with corresponding biotinylated secondary antibodies. The chromogenic reaction was conducted with diaminobenzidine (Zhongshan Biosciences, Beijing, China) for 3 to 5 min and terminated by rinsing with water. The sections were subsequently stained with hematoxylin. Immunohistochemistry was performed on four-to-five pregnant mice from each group and each sample was assayed three times. The VEGFA, VEGFR2, and PLGF protein localization was analyzed and quantified using Medical Image Analysis Software (Beihang University, Beijing, China) and a yellowish-brown stain was determined as positive.

### 2.5. Western Blotting

Proteins were extracted from 40 mg decidual tissue collected at various time points during the post-implantation period from the two groups using a cell lysis buffer for western blotting and immunoprecipitation (Beyotim, Shanghai, China). Protein concentration was determined using the bicinchoninic acid Protein Assay kit (Beyotim, Shanghai, China). Samples were boiled in 5× sodium dodecylsulfate (SDS) sample loading buffer for 10 min and then loaded onto a 10% SDS-polyacrylamide gel (Beyotim, Shanghai, China). Following electrophoresis, proteins were transferred onto polyvinylidene difluoride membranes (Bio-Rad, California, USA). The membranes were blocked for 80 min at room temperature in a Tris Buffered Saline with Tween (TBST) buffer (20 mM Tris (pH 7.6), 137 mM NaCl, and 0.05% (w/v) Tween 20) containing 5% non-fat milk. Immunoblotting was performed by incubating the membranes in 5% milk-TBST overnight at 4 °C with a rabbit monoclonal anti-VEGFA (ab52917, Abcam, Shanghai, China) antibody at a 1:500 dilution, a rabbit polyclonal anti-VEGFR2 (07-158, Abcam, Millipore, Billerica, USA) antibody at a 1:500 dilution, a rat monoclonal anti-PLGF (ab51654, Abcam, Shanghai, China) antibody at a 1:500 dilution, a mouse monoclonal anti-β-actin (ab8226, Abcam, Shanghai, China) antibody at a 1:1000 dilution, a rabbit monoclonal anti-estrogen receptor α (ERα) (04-227, Millipore, Billerica, USA) antibody at a 1:500 dilution, and a mouse monoclonal anti-progesterone receptor (PR) (ab2765, Abcam, Shanghai, China) antibody at a 1:500 dilution. Membranes were washed three times with TBST followed by incubation with the corresponding secondary antibody at room temperature for 80 min. After washing three times with TBST, positive bands were detected by enhanced chemiluminescence reagents (Beyotim, Shanghai, China) and quantified by densitometry using Quantity One version 4.4.0 software. Western blotting was performed on four-to-five pregnant mice from each group, and each sample was assayed three times.

### 2.6. Real-Time polymerase chain reaction (RT-PCR)

Total RNA was extracted from 30 mg decidual tissue using Trizol reagent (Invitrogen, Carlsbad, USA) according to the manufacturer’s instructions. cDNA synthesis was performed with 1 μg total RNA treated with DNase I in a 20 mL reaction system using the First Strand synthesis for RT-PCR kit (Takara, Dalian, China). cDNA was stored at −20 °C until real-time RT-PCR analysis. Specific primers for VEGFA, VEGFR2, PLGF, and β-actin were designed and produced by Sangon Biotech (Shanghai, China). The sequences of the primers used are shown in Table 1. To compare transcript levels of VEGFA, VEGFR2, and PLGF between the normal group and the folate-deficient group, real-time RT-PCR was carried out using SYBR Premix Ex Taq kits (Takara, Dalian, China) and a Bio-Rad CFX96 Real-Time System (Bio-Rad, California, USA). The real time RT-PCR master mixture (15 uL) consisted of 7.5 µL of 2× SYBR Premix Ex Taq, 0.6 µL of 10 pmol/mL primers, 1.2 µL of cDNA, and 5.1 µL of double-distilled H_2_O according to the manufacturer’s recommendations. The PCR conditions were as follows: initial denaturation at 94 °C for 30 s; 40 cycles of 10 s at 94 °C (denaturation), and 30 s at corresponding primer melting temperature (Tm). Relative gene expression levels were calculated with the 2^−ΔΔ*C*t^ method [19]. Real time RT-PCR was performed on six pregnant mice from each group and each sample was assayed three times.

**Table 1:** Sequences of forward and reverse primers used in real-time RT-PCR (5′→3′).

Gene	Forward	Reverse
VEGFA	GTCCAACTTCTGGGCTCTTCT	CCCTCTCCTCTTCCTTCTCTTC
VEGFR2	TGGCAAATACAACCCTTCAGAT	GTCACCAATACCCTTTCCTCAG
PLGF	GCCGATAAAGACAGCCAACA	CATTCACAGAGCACATCCTGA
β-actin	CCTGAGGCTCTTTTCCAGCC	TAGAGGTCTTTACGGATGTCAACGT

VEGFA, vascular endothelial growth factor A; VEGFR2, vascular endothelial growth factor receptor 2; PLGF, placental growth factor.

### 2.7. Statistical Analysis

Data were analyzed using the Statistical Package for the Social Sciences (SPSS) statistical software (version 16.0, SPSS, Chicago, USA). Values are given as the mean ± SEM. The differences between groups in serum levels of folate, Hcy, P4, and E2 and the expression of VEGFA, VEGFR2, PLGF, and PR were analyzed with Student’s *t*-test. Bonferroni correction was used for multiple testing, while the differences between groups in serum levels of FSH, LH, and PRL were analyzed with two-way analysis of variance. A *p* < 0.05 was considered statistically significant.

## 3. Results 

### 3.1. Validation of the Folate-Deficient Pregnant Mouse Model

The electro-chemiluminescence immunoassay was used to measure the serum folate levels to verify the validity of the pregnant mouse model. As shown in Table 2, serum folate levels were significantly decreased in the folate-deficient group (*p* < 0.01), which was consistent with our previous study [20]. In addition, Hcy, the intermediate product in folate metabolism and one of the factors influencing the biological functions of the vascular endothelium, serum levels were also determined with ELISA. As shown in Table 3, serum Hcy levels were significantly increased in the folate-deficient group (*p* < 0.01).

**Table 2:** Serum folate levels (ng/mL) in the pregnant mice (mean ± standard error of the mean (SEM)).

	Normal (*n*)	Folate-deficient (*n*)	*p*
**Serum folate**	>20 (*10*)	4.83 ± 0.48 (*19*)	<0.01

**Table 3:** Serum Hcy levels (μmol/L) in the pregnant mice (mean ± standard error of the mean (SEM)).

	Normal (*n*)	Folate-deficient (*n*)	*p*
E6	7.50 ± 0.24 (*5*)	11.06 ± 0.40 (*5*)	<0.01
E7	6.75 ± 0.41 (*5*)	12.05 ± 0.57 (*5*)	<0.01
E8	8.26 ± 0.32 (*5*)	12.46 ± 0.61 (*5*)	<0.01

E6, Embryonic day 6; E7, Embryonic day 7; E8, Embryonic day 8.

### 3.2. Decidual Angiogenesis Was Restrained in Folate-Deficient Pregnant Mice

**Figure 1:**
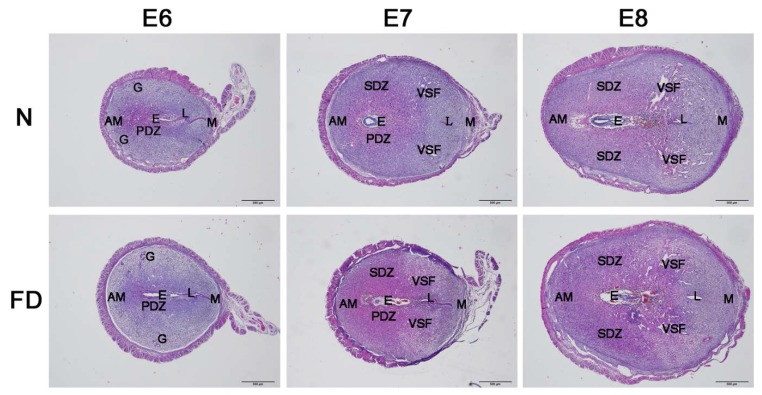
Uterine section’s hematoxylin-eosin staining from E6 to E8. No significant abnormal decidualization or embryonic development occurred in the folate-deficient group. N: normal group; FD: folate-deficient group; E: embryo; L: luminal epithelium; G: glandular epithelium; PDZ: primary decidual zone; SDZ: secondary decidual zone; M: mesometrial; AM: anti-mesometrial; VSF: vascular sinus folding; Scale bar: 500 μm; E6, Embryonic day 6; E7, Embryonic day 7; E8, Embryonic day 8.

**Figure 2:**
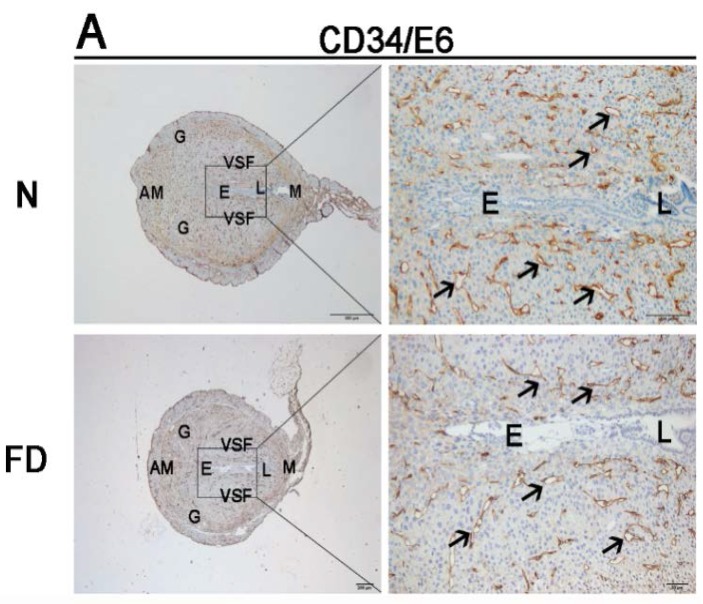

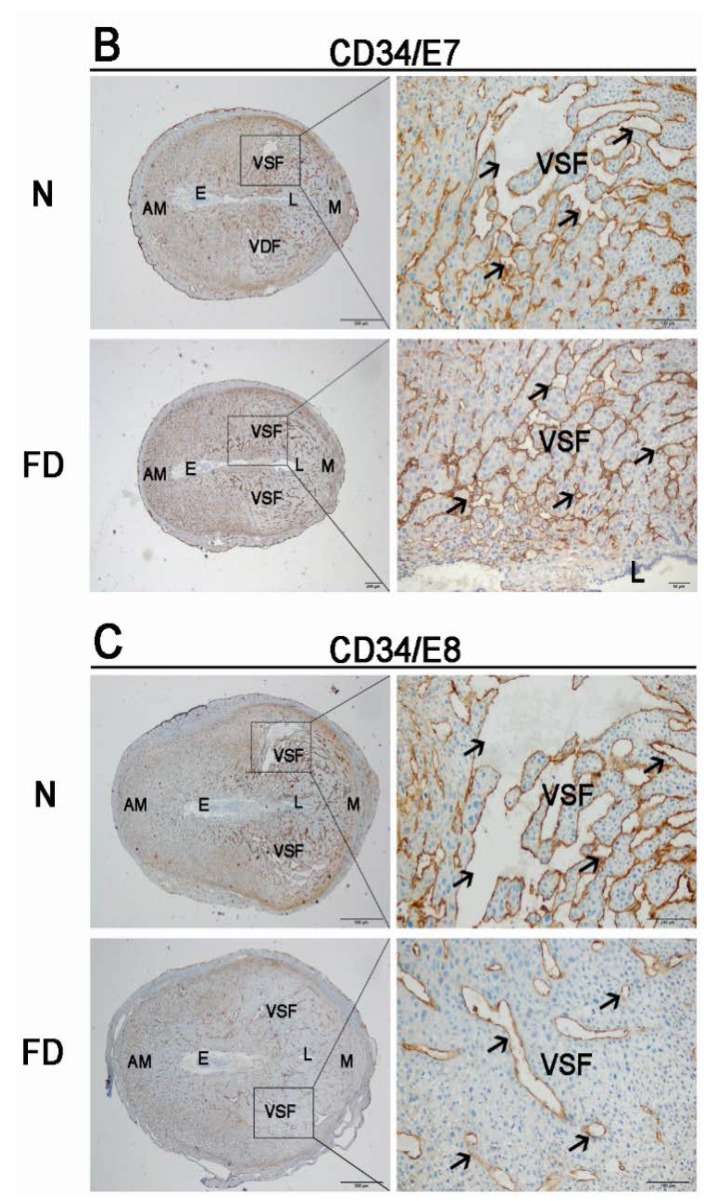
Immunohistochemistry staining with cluster designation 34 (CD34) from Embryonic day 6 (E6) to Embryonic day 8 (E8). Impaired formation of decidual angiogenesis was detected in the folate-deficient group. The pictures in the right column are the higher magnification images of the black boxes in the left column. (**A**) The magnified area was the central region around the embryo in uterus at E6. (**B**, **C**) The magnified area was one side of VSF at E7 and E8. Arrow indicates the variable-sized blood vessels. N: normal group; FD: folate-deficient group; E: embryo; L: luminal epithelium; G: glandular epithelium; M: mesometrial; AM: anti-mesometrial; VSF: vascular sinus folding. Scale bar: 500 μm (left), 100 μm (right).

To determine whether there was any abnormal angiogenesis in the folate-deficient group, the structural changes in blood vessels, the molecular regulation of angiogenesis, and the vascular remodeling in the uterus were analyzed from E6 to E8, which was after implantation (E4–5) but prior to initial placenta establishment (E9–10). As illustrated in the H&E staining, no obvious abnormalities could be seen in the folate-deficient group (Figure 1). With the development of the process of pregnancy, there was no significant difference in morphology and structure in the two groups. In the folate-deficient mice, the blastocyst could implant in the mesangial contralateral visibly. Stroma cell decidulization occurred around the blastocyst continuously and formed the primary decidual zone and secondary decidual zone gradually. The development of the blastocyst was normal as well. However, the significant abnormalities were observed in the vascular density and the enlargement and elongation of vascular sinus folding in folate-deficient pregnant mice, as evidenced with CD34 staining from E6 to E8 (Figure 2). Normally, variable-sized vascular sinus foldings [21] are distributed symmetrically in the central region of the uterus and are enlarged and elongated over time, whereas fine mesh-like blood vessels are arrayed in the anti-mesometrial region, which was similar to our findings (Figure 2). Closer observation of the central region revealed that the sprouting process was most active at E6 (Figure 2A), whereas enlargement and elongation of vascular sinus folding and intussusception of blood vessels [22] were dominant from E7 to E8. Time-series analyses indicated that, compared with E6, the number of large-sized vascular sinus foldings in the central region as well as blood vessel densities in the anti-mesometrial region were markedly increased at E7 and E8 (Figure 2B,C). A significant decrease in vascular density was observed in the folate-deficient group from E6 to E8 as compared with the normal group (Figure 2). The vascular sinus foldings, which predominantly extend into the intermediate zone from the mesometrial to the anti-mesometrial region of the decidua, appeared to be diminished in the folate-deficient group as compared with the normal group, especially at E7 and E8 (Figure 2B,C).

### 3.3. The Expression of VEGFA, VEGFR2, and PLGF Was Reduced in Folate-Deficient Decidual Tissue

**Figure 3:**
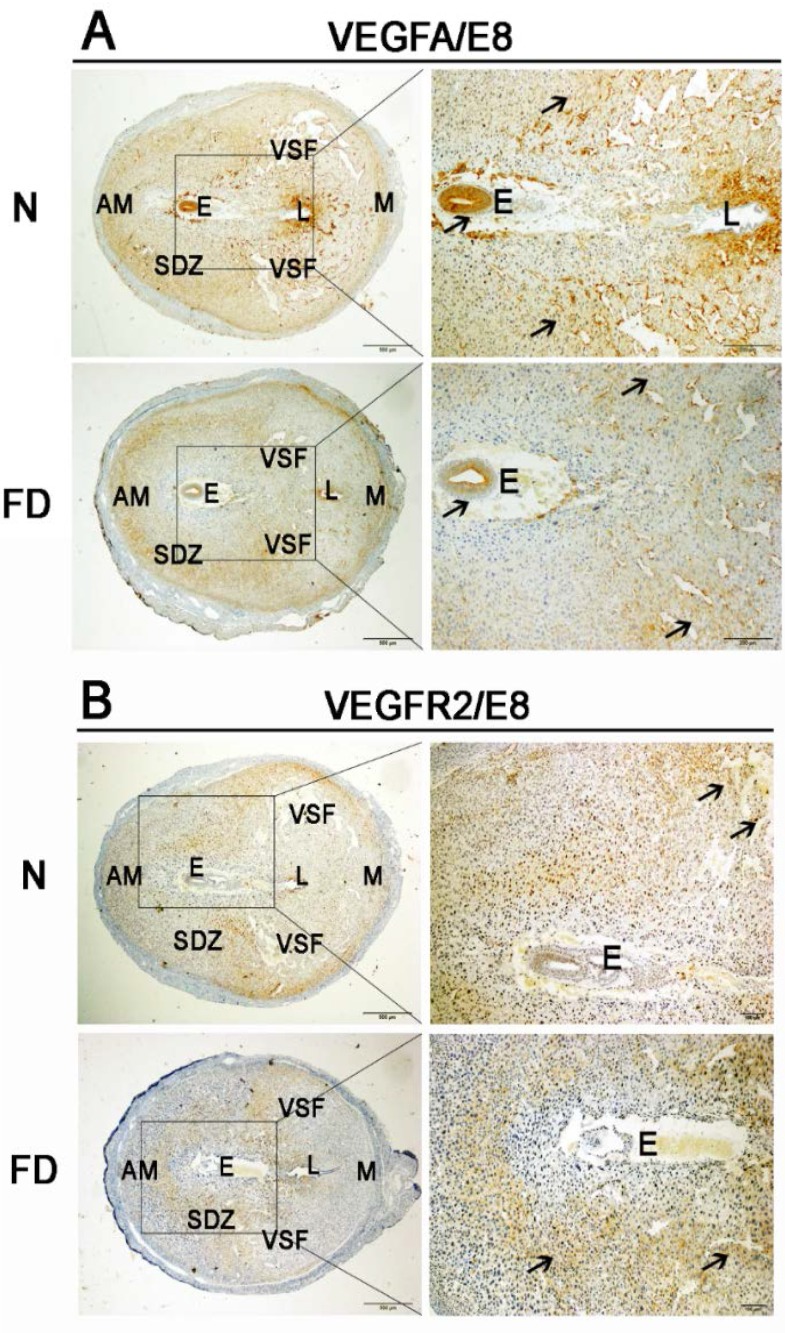

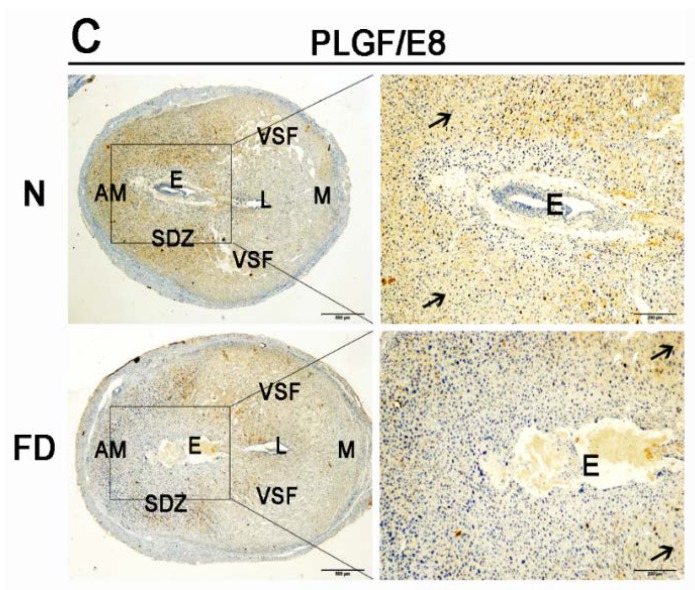
Immunohistochemistry staining with vascular endothelial growth factor A (VEGFA), vascular endothelial growth factor receptor 2 (VEGFR2), and placental growth factor (PLGF). Decreased expression of VEGFA, VEGFR2, and PLGF was detected in the folate-deficient group. The pictures in the right column are the higher magnification images of the black boxes in the left column. Additionally, the black boxes were the uterus’s central region around the embryo. Arrow indicates each factor’s different expression and distribution between two groups. (**A**) VEGFA was localized to a wide area of the secondary decidual zone (PDZ) and the embryo in both normal and folate-deficient mice, but there was decreased expression in the folate-deficient group. (**B**) VEGFR2 was localized mainly to the VSF close to the AM region in normal mice, whereas VEGFR2 was localized mainly to part of the PDZ in folate-deficient mice. (**C**) PLGF had a similar localization pattern as VEGFA in normal mice but was not expressed in the embryo. However, PLGF was expressed mainly in the central region of the uterus instead of in the PDZ in folate-deficient mice. N: normal group; FD: folate-deficient group; E: embryo; L: luminal epithelium; SDZ: secondary decidual zone; M: mesometrial; AM: anti-mesometrial; VSF: vascular sinus folding. Scale bar: 500 μm (left), 200 μm (right).

**Figure 4:**
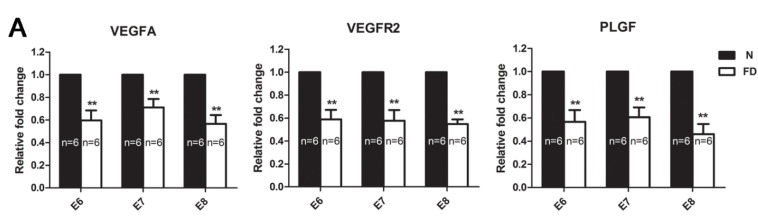

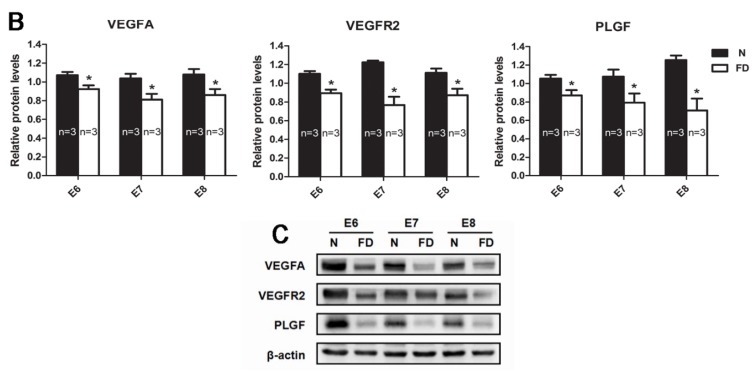
Real-time polymerase chain reaction (RT-PCR) and western blot analysis of the expression of vascular endothelial growth factor A (VEGFA), vascular endothelial growth factor receptor 2 (VEGFR2), and placental growth factor (PLGF) from Embryonic day 6 (E6) to Embryonic day 8 (E8). (**A**) The mRNA levels of VEGFA, VEGFR2, and PLGF in folate-deficient mice were significantly decreased compared with normal mice from E6 to E8 (** *p* < 0.01). Data are presented as the mean ± standard error of the mean (SEM). (**B**) Protein levels in folate-deficient mice were compared with normal mice. Statistical analysis of protein expression was from three independent experiments (* *p* < 0.05). Data are presented as the mean ± SEM. (**C**) Western blot analysis of three angiogenic growth factors. β-actin was used as a loading control. The protein levels of VEGFA, VEGFR2, and PLGF showed similar trends to the mRNAs levels. N: normal group, FD: folate-deficient group.

Immunohistochemical staining for VEGFA, PLGF, and VEGFR2 in the decidual tissue on E8 is shown in Figure 3. VEGFA was distributed mainly in a wide area of the secondary decidual zone (PDZ) and embryo in both normal and folate-deficient mice, but the expression of VEGFA in folate-deficient mice was depressed (Figure 3A). VEGFR2 was localized mainly in the vascular sinus folding close to the anti-mesometrial region in normal mice, whereas VEGFR2 surrounded the embryo mainly in folate-deficient mice (Figure 3B). The localization of PLGF was similar to VEGFA in normal mice, but it did not distribute in the embryo. However, PLGF was mainly located in the central region of the uterus instead of the PDZ in folate-deficient mice (Figure 3C). The relative amounts of VEGFA, PLGF, and VEGFR2 mRNAs detected by real-time RT-PCR are shown in Figure 4A. The relative fold change of VEGFA, PLGF, and VEGFR2 mRNA in the folate-deficient mice was decreased compared with the levels in normal mice from E6 to E8 (*p* < 0.01). The protein expression levels of VEGFA, PLGF, and VEGFR2 analyzed by Western blotting were correlated with the level of mRNAs (Figure 4B).

### 3.4. Folate-Deficient Mice Showed Disordered Reproductive Hormone Levels 

**Figure 5:**
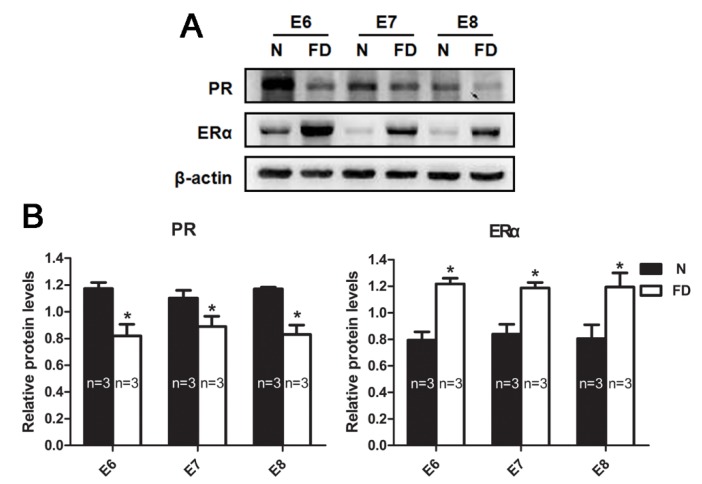
Western blot analysis of the expression of progesterone receptor (PR) and estrogen receptor α (ERα) from Embryonic day 6 (E6) to Embryonic day 8 (E8). (**A**) Protein levels are expressed relative to the normal group and β-actin was used as a loading control. (**B**) Statistical analysis of protein expression from three independent experiments. (* *p* < 0.05). PR was significantly decreased in the folate-deficient group compared with the normal group, whereas ERα was elevated significantly in folate-deficient mice. β-actin was used as a loading control. N: normal group; FD: folate-deficient group.

**Figure 6:**
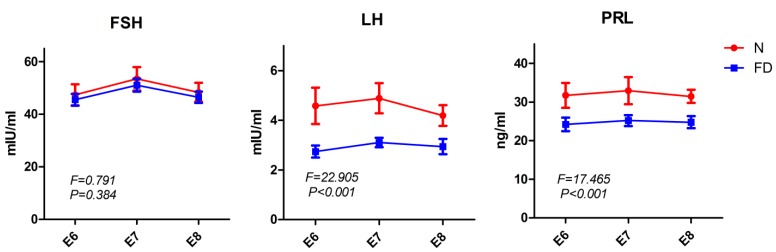
Serum follicle stimulating hormone (FSH), luteinizing hormone (LH) and prolactin (PRL) levels in the pregnant mice. Serum LH (*F* = 22.905, *p* < 0.001) and PRL (*F* = 17.465, *p* < 0.001) levels were decreased in the folate-deficient group significantly, but the level of FSH (*F* = 0.791, *p* = 0.384) was not significantly different between the two groups. Data are presented as the mean ± standard error of the mean (SEM). N: normal group; FD: folate-deficient group.

Correct steroid hormone levels characterize a successful pregnancy, including decidualization, angiogenesis, and placentation. Angiogenesis could be impaired by abnormal steroid hormone levels [23]. To further assess the effect of folate deficiency on steroid hormone levels, we measured the serum levels of P4 and E2 from E6 to E8 by ELISA. As shown in Table 4, folate-deficient mice presented a decreasing trend in serum levels of P4 and E2 compared with normal mice. Western blotting demonstrated that the protein expression of PR in decidual tissue was decreased from E6 to E8, whereas the protein expression of ERα was increased in the folate-deficient group compared with the normal group (Figure 5). Because the secretion of P4 and E2 is regulated by gonadotropins, including FSH, LH and PRL, we also detected these three gonadotropins at the same time. The results showed that folate-deficient mice had aberrant gonadotropin levels (Table 5, Figure 6). Although there was no significant difference in the serum FSH levels between the two groups (*F* = 0.791, *p* = 0.384), the folate-deficient mice had a relatively lower serum level of LH (*F* = 22.905, *p* < 0.001) and PRL (*F* = 17.465, *p* < 0.001) compared with the normal mice (Figure 6). 

**Table 4:** Serum P4 and E2 levels in the pregnant mice (mean ± standard error of the mean (SEM)).

	P4 (ng/mL)			E2 (pmol/L)		
	Normal (*n*)	Folate-deficient (*n*)	*p*	Normal (*n*)	Folate-deficient (*n*)	*p*
E6	11.02 ± 0.44 (*4*)	8.79 ± 0.17 (*4*)	0.009	42.39 ± 0.82 (*3*)	29.97 ± 0.60 (*4*)	<0.001
E7	10.02 ± 0.21 (*4*)	7.56 ± 0.18 (*4*)	<0.001	38.01 ± 0.77 (*3*)	26.50 ± 0.65 (*4*)	<0.001
E8	10.24 ± 0.16 (*4*)	6.20 ± 0.26 (*4*)	<0.001	38.76 ± 0.71 (*3*)	23.26 ± 0.71 (*4*)	<0.001

P4, progesterone; E2, estradiol.

**Table 5:** Serum FSH, LH, and PRL levels in the pregnant mice (mean ± standard error of the mean (SEM)).

	FSH (mIU/mL)		LH (mIU/mL)		PRL (ng/mL)	
	Normal (*n*)	Folate-deficient (*n*)	Normal (*n*)	Folate-deficient (*n*)	Normal (*n*)	Folate-deficient (*n*)
E6	47.38 ± 4.02 (*3*)	45.50 ± 2.23 (*5*)	3.72 ± 0.51 (*3*)	2.66 ± 0.15 (*5*)	31.74 ± 3.23 (*3*)	24.21 ± 1.76 (*5*)
E7	53.49 ± 4.43 (*3*)	51.07 ± 2.46 (*5*)	4.52 ± 0.60 (*3*)	3.28 ± 0.20 (*5*)	32.94 ± 3.53 (*3*)	25.21 ± 1.42 (*5*)
E8	48.36 ± 3.62 (*3*)	46.45 ± 2.19 (*5*)	3.88 ± 0.45 (*3*)	2.95 ± 0.21 (*5*)	31.46 ± 1.70 (*3*)	24.78 ± 1.55 (*5*)

FSH, follicle stimulating hormone; LH, luteinizing hormone; PRL, prolactin.

## 4. Discussion 

In humans, the influence of folate nutritional status on various outcomes of a pregnancy has long been recognized. Although fetuses are known to concentrate folate from the maternal circulation to fulfill their nutrient requirements, it has been demonstrated in several animal models, including rat [24], monkey [25], golden hamster [26], and mouse [27], that severe maternal folate deficiency in the preconception and gestational periods may hamper female fertility and embryo and fetal viability. These animal findings emphasize that folate is indispensable during mammalian folliculogenesis and fetal development. However, much less is known about the mechanism of the folate deficiency on reproduction. Our previous study showed that a five-week folate deficiency status did not influence embryo implantation, and the methylation and expression of two molecules (cadherin 1 and progesterone receptor) essential for uterine receptivity were not altered either [13]. However, the outcome of the folate-deficient pregnant mice was not favorable (supplementary information). It is well known that successful implantation does not necessarily mean a successful pregnancy. After implantation, the normal uterine decidualization, placentation, and a healthy embryo are all crucial for pregnancy. Decidual angiogenesis forms a new vascular network that serves as the first exchange apparatus between the maternal circulation and the embryo that is necessary for embryonic survival and a successful pregnancy. Therefore, in this study, we focused on decidual angiogenesis, which occurs shortly after interstitial implantation. 

In rodents and humans, the implanting conceptus stimulates decidualization in a specific spatial-temporal pattern [28]. The decidual reaction occurs during early pregnancy when the fibroblast-like endometrial stromal cells transiently proliferate and then differentiate into large, polyploid decidual cells. However, no significant abnormalities could be seen in the folate-deficient group as evidenced by the hematoxylin-eosin staining in our study. In response to the implanting blastocyst, neoangiogenesis establishes a network of vessels and sinusoids within the compact decidual tissue to serve as the first exchange apparatus between the maternal circulation and the developing embryo [29]. In the normal group, closer observations of the central region revealed that the sprouting process was most active at E6, and enlargement and elongation of vascular sinus folding and intussusception of blood vessels were dominant from E7 to E8. However, compared with the normal group, a significant decrease in vascular density was observed in the folate-deficient group from E6 to E8. The vascular sinus foldings, which predominantly extend into the intermediate zone from the mesometrial to the anti-mesometrial region of the decidua, also appeared to be diminished at E7 and E8 in the folate-deficient group. Taken together, these results indicate that folate deficiency could restrain decidual angiogenesis in pregnant mice.

The VEGF family is important in regulating decidual angiogenesis and maternal spiral artery remodeling [29]. The endometrium, decidua, and placenta are rich sources of angiogenic growth factors [30]. Several studies have reported the expression of VEGFA and its receptors in first-trimester human decidua, including in endothelial cells, epithelial cells, macrophages, and trophoblasts [31,32]. Therefore, VEGFA seems to play an active role in trophoblast invasion and angiogenesis during implantation. Though VEGFA can play several roles with different receptors, some researchers [15,33] demonstrated that mouse decidual angiogenesis could be blocked by interference with the vascular endothelial growth factor VEGFA/VEGFR2 pathway, the main player in initiating sprouting angiogenesis. PLGF is abundantly expressed in the human placenta, with expression increasing from the first trimester to the late second trimester and subsequently declining until delivery [34]. It is expressed in villous and extravillous trophoblast cells, vascular endothelium, and decidual stromal cells, and may act as a mediator of trophoblast function and angiogenesis during early pregnancy [35]. PLGF is known to mediate the formation of a mature and stable vessel network, which is an important feature in facilitating and resisting the dramatic increase in blood supply at the implantation site to serve the growing demands of the fetus [36]. PLGF and VEGFA have synergistic effects in the induction of angiogenesis, but PLGF-induced vessels are more mature and stable than VEGF-induced vessels [37,38]. In our study, a reduction of these three factors (VEGFA, VEGFR2, and PLGF) was accompanied by the damage of decidual angiogenesis in pregnant mice during the post-implantation period. It revealed that the abnormal expression and distribution of VEGFA, VEGFR2, and PLGF resulted in abnormal decidual angiogenesis in folate-deficient pregnant mice.

Previous studies have found that Hcy has vasculotoxic properties, causing endothelial cell damage and dysfunction [39,40]. Hcy is also known to induce oxidative stress responses [39] and to decrease the cellular antioxidant potential [40], which can damage all components of the endothelial cell. To explore why the decidual angiogenesis was impaired in folate-deficient mice, we tested the serum Hcy levels during the post-implantation period. It is known that dietary or genetically determined folate deficiency leads to Hcy accumulation [41] because both the remethylation and transsulfuration [42] pathways are inhibited in folate deficiency. We found a significant increase in the serum Hcy levels in the folate-deficient group compared with the normal group. When endothelial cells are exposed to Hcy *in vitro*, protein misfolding may be induced in the endoplasmic reticulum by altering local redox potential and interfering with disulfide bond formation [43]. Accumulation of misfolded proteins in the endoplasmic reticulum can trigger an unfolded protein response [44], which may cause cellular growth arrest [40] and apoptosis [45] if the endoplasmic reticulum stress is prolonged. In addition, Hcy exposure causes impaired early extra-embryonic vascular development, as evidenced by the altered composition of the vascular beds as well as the reduced expression of VEGFA and VEGFR2 [46] and even reduced the expression of VEGFA in a dose-dependent manner *in vitro* [47]. These findings were in accordance with our results, which showed that the reduced expression of VEGFA, VEGFR2, and PLGF in folate-deficient pregnant mice impacts decidual angiogenesis and may be associated with the increased serum Hcy levels.

Ovarian steroid hormones play a pivotal role in directing early uterine events during pregnancy, including make the uterus competent to attach to the blastocyst and initiate the process of implantation [48,49]. Subsequently, ovarian steroid hormones regulate a series of complex interactions at the interface between the developing embryo and the cells in the stromal compartment, leading to the formation of decidua, which supports embryo growth and maintains early pregnancy [50]. The cellular actions of these hormones are mediated through intracellular estrogen receptor and progesterone receptor proteins, which are hormone-inducible transcription factors [51]. The receptors were known to bind their respective hormone, to bind to DNA, and to regulate specific gene transcription. These genomic actions trigger the expression of specific gene networks in different cell types within the uterus, and the products of these genes in turn mediate the pregnancy. Angiogenesis also could be impaired by abnormal ovarian steroid hormone levels. Two decades ago, Keshet *et al.* first suggested that high VEGFA expression in decidual stromal cells could be regulated by ovarian steroid hormones [52]. Since then, the roles of P4, E2, and their receptors in decidual angiogenesis have been studied. Both P4 and E2 have been shown to induce VEGF expression in human uterine stromal cells [53,54]. In this study, the folate-deficient mice presented disordered ovarian steroid hormone levels and their receptors’ expression was also abnormal compared with normal mice. Western blotting showed significant down-regulation of PR and up-regulation of ERα in folate-deficient groups, respectively. We detected a low level of E2 in folate deficiency in pregnant mice during E6 to E8, but there was a significant increase in ERα expression compared with the control group. The inconsistency between levels of this hormone and its receptor has been noted in several *in vivo* studies involving drug treatments or others [55]. The strength of the effect of hormones is related to the number of the hormone-receptor complex, so keeping the appropriate number of this complex is necessary to maintain the organism’s normal functions. Either serum hormone level or the hormone receptor has a quantitative/qualitative change that could result in endometrial lesions. Hormone-activated receptors exert a feedback regulatory effect on the transcription of their own parent genes. Thus, the decreased downstream products may be responsible for the increased expression of ERα. Certainly we need to research it further to verify this hypothesis. In addition, Gao’s research showed that the ERα promoter was significantly less methylated in the folate-deficient tissues compared with the normal ones [13]. The increased expression of ERα in decidual tissue may be due to the low level of methylation of ERα. However, no matter which reason led to increased expression of ERα, the E2-activated ERα complex was reduced authentically in folate-deficient pregnant mice and the complex’s function was getting weak. Therefore, we speculated that folate deficiency may disturb the balance between P4 and E2, as well as their respective receptors. Additionally, the reduction of VEGFA, VEGFR2, and PLGF may be connected with this disordered balance.

In addition, it is known that reproductive fecundity depends on the coordinated functions of organs and glands along the hypothalamic-pituitary-gonadal axis. Gonadoptropin are synthesized in the pituitary gland and induce the ovaries to produce P4 and E2. The main function of FSH is to stimulate ovarian growth and promote follicular development [56]. LH participates in ovarian regulation, plays a critical role in follicular maturation, ovulation, and corpus luteum development, and intervenes in the synthesis of steroid hormones [57]. PRL secretion induced by mating leads to increased endothelial cell proliferation in the corpus luteum of pregnant rodents and plays a critical role in corpus luteum maintenance, which promotes progesterone production and the maintenance of gestation [58]. To investigate whether disruption of the pituitary regulation of the ovary might be responsible for the reduction in steroid hormone levels, we also measured serum gonadotropins, including FSH, LH, and PRL. There was no significant difference in serum FSH levels between the two groups; however, folate-deficient mice had lower serum levels of LH and PRL compared with normal mice in our study, indicating that the effect of folate deficiency on hypothalamic-pituitary-gonadal axis function was likely to be causal in the disordered steroid hormone levels. Moreover, P4 and E2 are cholesterol-derived, phylogenetically old steroid hormones [59]. They are synthesized during steroid hormone metabolization within several cell types such as the corpus luteum and placenta [60]. In a recent review [61], a novel insight into the interactions between folate and the lipid metabolism was put forward. The folate-deficient diet led to steatosis by altering the balance of phospholipids, including phosphatidylcholine, phosphatidylethanolamine, sphingomyelin, and components of very low-density lipoprotein (VLDL) that transport lipids from the liver. In addition, Hcy and the lipid metabolism are interrelated, at least partly, via methyl group metabolism [62]. It was found that lipid metabolism-related genes and associated metabolic pathways were regulated extensively by folate deficiency. These findings may explain our results of disordered steroid hormone levels in folate-deficient mice but still leave much to be explored in future efforts. 

In conclusion, folate deficiency could impair decidual angiogenesis via the down-regulation of pro-angiogenic factors including VEGFA, VEGFR2, and PLGF, which results in a poor vascular network. In addition, the vasculotoxic properties of Hcy and abnormal reproductive hormone levels may contribute to the impaired decidual angiogenesis. Our previous published study had enriched the awareness that folate deficiency disrupts the proliferation-apoptosis balance and that the mitochondrial apoptosis pathway of endometrial decidual cells was inhibited in folate-deficient pregnant mice. Additionally, the marker genes’ expression of endometrium decidualization in mice, including bone morphogenetic protein 2 (BMP2), homeobox A 10 (Hoxa10), matrix metalloproteinase 2 (MMP2), and matrix metalloproteinase 9 (MMP9) proteins was markedly reduced in folate-deficient mice. Combining all the evidence, we reach the final conclusion that folate deficiency could effect the uterine decidualization and decidual angiogenesis of pregnant mice. This research may provide some theories and evidence for investigating the mechanism underlying poor reproduction in females that have poor folate intake and absorption. 

## 5. Conclusions

Folate deficiency could impair decidual angiogenesis via the down-regulation of pro-angiogenic factors including VEGFA, VEGFR2, and PLGF, which results in a poor vascular network. In addition, the vasculotoxic properties of Hcy and abnormal reproductive hormone levels may contribute to the impaired decidual angiogenesis. 

## Supplementary Files

Supplementary File 1
