# Supplementary material for: Folate Deficiency Could Restrain Decidual Angiogenesis in Pregnant Mice
Source: Nutrients. 2015 Aug 4;7(8):6425–45. doi: 10.3390/nu7085284 (PMC4555123; doi:10.3390/nu7085284)
Supplement: Supplementary File 1 [file nutrients-07-05284-s001.docx]

**Supplementary Information**


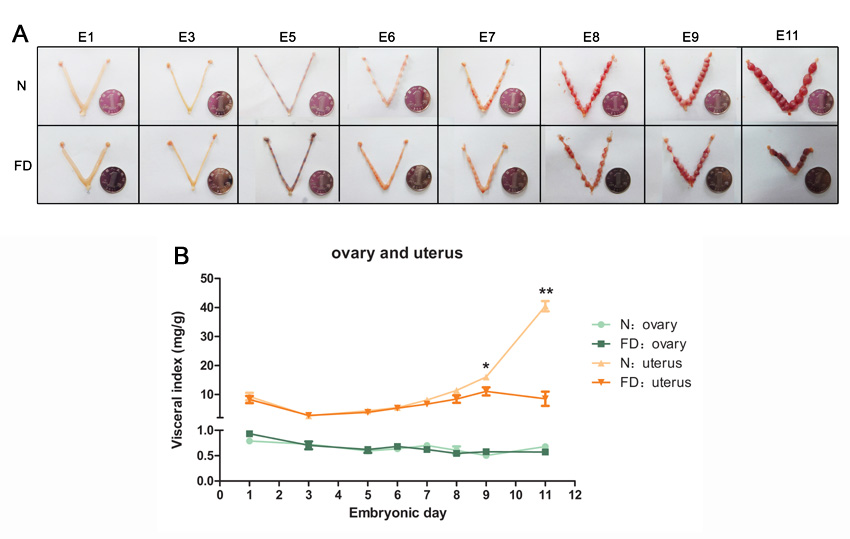


**Figure S1.** Impaired embryo development was detected in the folate-deficient group after Embryonic day 9 (E9). (**A**) Intact uteri containing embryos from Embryonic day 1 (E1) to Embryonic day 11 (E11). The diameter of the coin is 19.5 mm. (**B**) [Visceral](javascript:void(0);) [index](javascript:void(0);) of ovary and uterus from E1 to E11. N: normal group; FD: folate-deficient group
(* *p* < 0.05, ** *p* < 0.01).


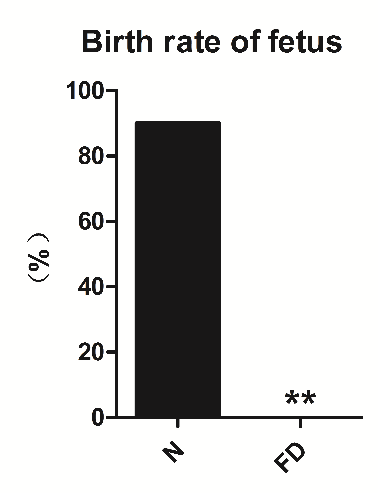


**Figure S2.** Folate deficiency results in adverse pregnancy. The birth rate of fetus in folate-deficient group is 0 (0/20), while the normal group is 90% (18/20). N: normal group, FD: folate-deficient group. (** *p* < 0.01)
